# Solid-Phase Synthesis of Well-Defined Multiblock Copolymers by Atom Transfer Radical Polymerization

**DOI:** 10.1021/jacs.4c03675

**Published:** 2024-07-30

**Authors:** Grzegorz Szczepaniak, Kriti Kapil, Samuel Adida, Khidong Kim, Ting-Chih Lin, Gorkem Yilmaz, Hironobu Murata, Krzysztof Matyjaszewski

**Affiliations:** † Department of Chemistry, Carnegie Mellon University, 4400 Fifth Avenue, Pittsburgh, Pennsylvania 15213, United States; ‡ Faculty of Chemistry, University of Warsaw, Pasteura 1, 02-093 Warsaw, Poland

## Abstract

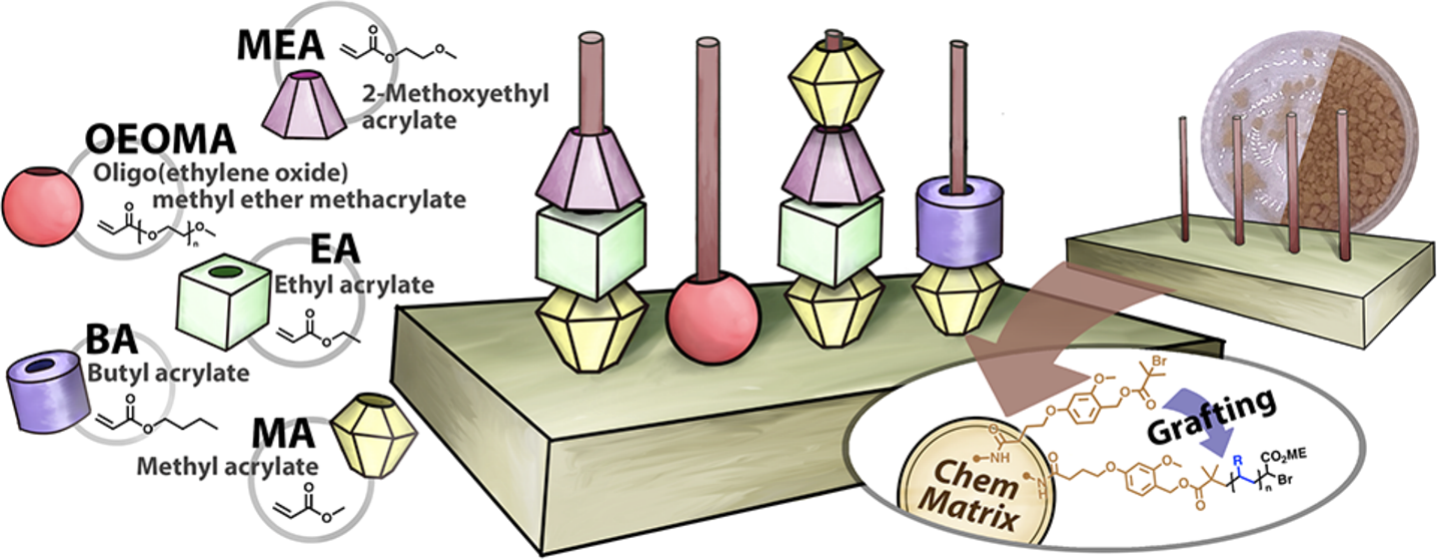

Solid-phase polymer synthesis, historically rooted in peptide synthesis, has evolved into a powerful method for achieving sequence-controlled macromolecules. This study explores solid-phase polymer synthesis by covalently immobilizing growing polymer chains onto a poly(ethylene glycol) (PEG)-based resin, known as ChemMatrix (CM) resin. In contrast to traditional hydrophobic supports, CM resin’s amphiphilic properties enable swelling in both polar and nonpolar solvents, simplifying filtration, washing, and drying processes. Combining atom transfer radical polymerization (ATRP) with solid-phase techniques allowed for the grafting of well-defined block copolymers in high yields. This approach is attractive for sequence-controlled polymer synthesis, successfully synthesizing di-, tri-, tetra-, and penta-block copolymers with excellent control over the molecular weight and dispersity. The study also delves into the limitations of achieving high molecular weights due to confinement within resin pores. Moreover, the versatility of the method is demonstrated through its applicability to various monomers in organic and aqueous media. This straightforward approach offers a rapid route to developing tailored block copolymers with unique structures and functionalities.

## Introduction

Historically rooted in the synthesis of peptides, solid-phase synthesis involves the binding of substrates to solid support material and the step-by-step addition of various molecules.^1^ This technique has been adapted and extended to prepare diverse biopolymers, including peptides,^2^ oligonucleotides,^3,4^ polysaccharides,^5,6^ and various classes of synthetic polymers.^7−12^ Solid-phase polymer synthesis involves the assembly of polymers on a solid support, immobilizing the growing chains throughout the synthetic process. This technique facilitated the purification and isolation of intermediate products, streamlined the synthesis workflow, reduced environmental impact, and enabled the synthesis of complex and multifunctional macromolecules with exquisite precision. The synergy between solid-phase techniques and modern advances in automation, high-throughput screening, and computational design can open new avenues for the rapid development of intricate polymers with tailored structures and functionalities.^13−19^

The solid-phase polymer synthesis proceeds by attaching the initiator of the first monomer to a solid support, typically a resin or a bead, bypassing the traditional limitations of diffusion encountered in solution-phase synthesis.^20^ This not only enables the use of excess reagents to drive reactions to completion but also minimizes the formation of undesired byproducts. Furthermore, the immobilization of the growing polymer chains facilitates the purification process through simple filtration and washing steps, reducing the complexity and resources required for purification compared to solution-phase synthesis.^21^

In recent years, researchers have harnessed the potential of solid-phase techniques to address challenges that were previously difficult to overcome by using conventional methods. The design of polymers with controlled architectures, such as block copolymers,^22−29^ dendrimers,^30−34^ and peptide–polymer hybrids,^7^ has been made more accessible due to the controlled growth achievable in solid-phase synthesis. Our group also explored solid-phase synthesis of protein–polymer hybrids on reversible immobilization supports (PARIS)^35^ and nucleic acid-polymer conjugates^8,36^ and demonstrated the method to be effective, rapid, and simple for automation. Additionally, the integration of functional moieties,^37^ postpolymerization modifications,^38^ and the synthesis of sequence-defined polymers have become increasingly feasible, enabling the development of materials with unprecedented properties and applications.^39−43^

Resin solvation (swelling) is one of the most important considerations in solid-phase organic synthesis (SPOS). Poor swelling results in poor reaction site accessibility, leading to diminished reaction rates.^44^ Polystyrene-based supports have traditionally been used in solid-phase polymer synthesis.^45,46^ However, polystyrene is hydrophobic and swells only in organic solvents. This poses a challenge for the synthesis of biocompatible/bioactive polymers, as they aggregate and are sensitive to degradation in organic solvents. Additionally, residual, potentially toxic, organic solvents, can remain after purification.

**Figure 1:**
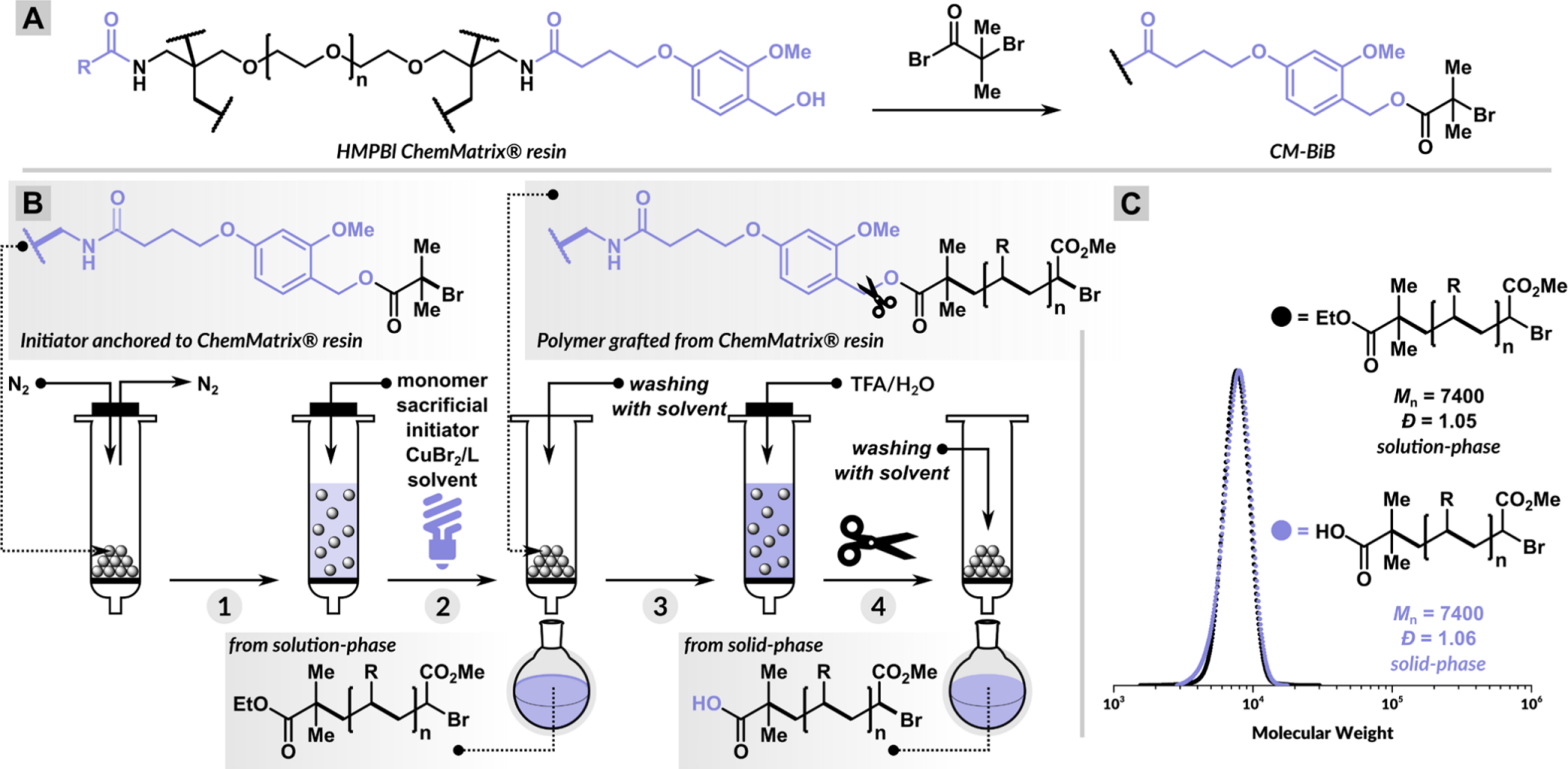
Solid-phase ATRP. (A) Synthesis of CM resin functionalized with the ATRP initiator (CM-BiB). Reaction conditions: BiB-Br,NEt_3_, CH_2_Cl_2_, rt, 3 h. (B) Solid-phase synthesis of poly(methyl acrylate) homopolymers. Reaction conditions: [MA]/[EBiB]/[CuBr_2_]/[Me_6_TREN] = 200/1/0.05/0.3, [CM-BiB] = 25 mg, in MeCN (50% *v*/*v*), irradiated for 3 h under UV light (370 nm, 7 mW/cm^2^) at ambient temperature, without monomer or solvent degassing. Reaction volume 5.0 mL. Procedure: (1) removal of air from syringe, (2) addition of monomer, sacrificial initiator, copper complex, solvent, (3) washing of resin with solvent, and (4) cleavage of polymer from resin. (C) SEC traces of PMA from the solution phase and the solid phase.

In this work, we have developed a robust approach for solid-phase synthesis of well-defined multiblock copolymers by ATRP via a “covalent approach” using a poly(ethylene glycol) (PEG)-based ChemMatrix resin (CM resin, Figure 1). The amphiphilic nature of PEG makes the resin well solvated in both aqueous and organic solvent media. As such, they can be easily filtered and washed with nearly any solvent. The CM resin exhibits loadings comparable to traditional polystyrene (PS) resins (0.4–0.7 mmol/g).^47^ Moreover, this resin transforms into a free-flowing state upon drying, simplifying the weighing process and minimizing material loss during transfer. Furthermore, the CM resin chain ends may be precisely functionalized. The functional groups allow for covalent modification of the resin to incorporate initiators suitable for controlled radical polymerization.

Reversible deactivation radical polymerization (RDRP) is an excellent tool for the synthesis of sequence-controlled polymers.^42,48−54^ Substantial work has been dedicated to spatial and temporal polymerization control via RDRP.^55−58^ Atom transfer radical polymerization (ATRP) is one of the most prevalent RDRP techniques that provides access to a diverse range of polymer architectures with high uniformity^59−65^ and retention of end-group functionality.^66^ ATRP is typically catalyzed by a transition metal complex, usually termed the activator [Cu^I^/L]^+^ (where L is a multidentate amine ligand), which reacts with an alkyl halide initiator (R–X), leading to the formation of a [X–Cu^II^/L]^+^, deactivator, and propagating radical (^•^R). Radical propagation occurs until the radical chain ends are deactivated by [X–Cu^II^/L]^+^, forming X-capped dormant species and regenerating [Cu^I^/L]^+^.^67−69^ This equilibrium favors a low concentration of active propagating species and a larger number of dormant chains.^70−73^ ATRP can be supplemented with various cocatalysts to afford temporal control^74−78^ and oxygen tolerance.^79−81^ The use of a small-molecule catalyst is particularly advantageous for transport within a confined system.^78,82^ However, previously reported solid-phase polymer synthesis immobilized on Wang resin (polystyrene -based) using traditional ATRP methods has reached limited success, especially for block copolymer synthesis.^83^

Our results demonstrate that the synergistic factors described above allow for a facile and sequence-controlled solid-phase polymer synthesis, resulting in di-, tri-, tetra-, and penta-block copolymers with excellent control over composition, molecular weight, and dispersity. Employing the fully oxygen-tolerant ATRP triggered by sodium pyruvate (SP), also known as photoinduced initiators for continuous activator regeneration (PICAR) ATRP, which is characterized by its rapid polymerization kinetics, quantitative conversions of monomers were achieved within 30 min of violet light (404 nm) irradiation.^84^ We highlighted the advantage of this methodology for the preparation of well-defined block copolymers by sequential grafting of polymer blocks at the living polymer chain ends, followed by efficient cleavage to obtain the final products. Additionally, we explored the limitations in achieving the highest molecular weight of polymers using this approach due to the confinement of the growing polymer chain within the pores of the resin. Finally, we broadened the scope of this technique to grafting various monomers in both organic and aqueous environments, thereby extending its versatility and applicability.

## Results and Discussion

### Synthesis of ATRP Initiator-Functionalized CM-BiB

HMPB ChemMatrix (CM) resin with benzylic hydroxyl groups (0.40–0.65 mmol/g) was functionalized with ATRP initiators by covalent modification of the hydroxy groups with α-bromoisobutyryl bromide (BiB-Br) in the presence of triethylamine in methylene chloride (Figure 1A). The resin was purified by multiple washes with the solvent and dried under vacuum for 48 h to obtain pure and dry CM-BiB (see the Supporting Information).

### Solid-Phase Polymer Synthesis

To verify the efficiency of polymerization from solid support, methyl acrylate (MA) was polymerized under classical photo-ATRP conditions using ethyl α-bromoisobutyrate (EBiB) as the sacrificial initiator (SI) in the solution phase,^86^ CM-BiB as the heterogeneous initiator in the solid-phase, and CuBr_2_/Me_6_TREN as the deactivator in acetonitrile (MeCN) (50% v/v with respect to MA) without prior deoxygenation (Figure 1B).

The use of SI molecules in the solution phase reduced the risk of terminating polymer chains growing from the CM-BiB, since a much smaller fraction of the CM-BiB initiator was involved in unwanted side reactions compared to when no SI was used. This increased control over the polymerization process, especially when polymerizations were conducted in the presence of air with low CM-BiB loading. In addition, the use of SI in excess allowed precise control over the final length of the polymer chains growing from the resin, even at low CM-BiB loadings. Furthermore, since the SI primarily determined the polymer chain length, the exact amount of CM-BiB used became less critical, simplifying the experimental setup and improving the reproducibility of the polymerization process. Finally, the presence of SI facilitated the monitoring of the polymerization process, especially during the synthesis of multiblock copolymers. The synthesis progress of each block was monitored independently by ^1^H NMR and SEC analyses of the homopolymers formed in the solution phase.

The polymerizations were carried out in solid-phase extraction (SPE) syringes equipped with 0.2 μm frits placed in a photoreactor with UV-LEDs (370 nm, 7 mW/cm^2^) at ambient temperature (Figure S2). After 3 h, the polymerization was stopped, and the solution phase was separated from the heterogeneous solid support. ^1^H NMR of the solution phase showed a 45% conversion of MA. SEC analysis of PMA revealed that the polymerization proceeded with good control over molecular weight and dispersity (*M*_n,SEC_ = 7400, *Đ* = 1.05) (Figure 1C). Further, the CM resin grafted with polymer was thoroughly washed with 10-fold MeCN and then subjected to TFA/H_2_O (95% *v*/*v*) to cleave the benzylic ester bond to release the immobilized PMA (Figure 1B). The thus-obtained polymer was analyzed by SEC. Interestingly, the PMA cleaved from the CM resin showed excellent agreement in molecular weight and dispersity with the solution-phase product and with the theoretical molecular weight based on the monomer conversion by ^1^H NMR (Figure 1C).

**Figure 2:**
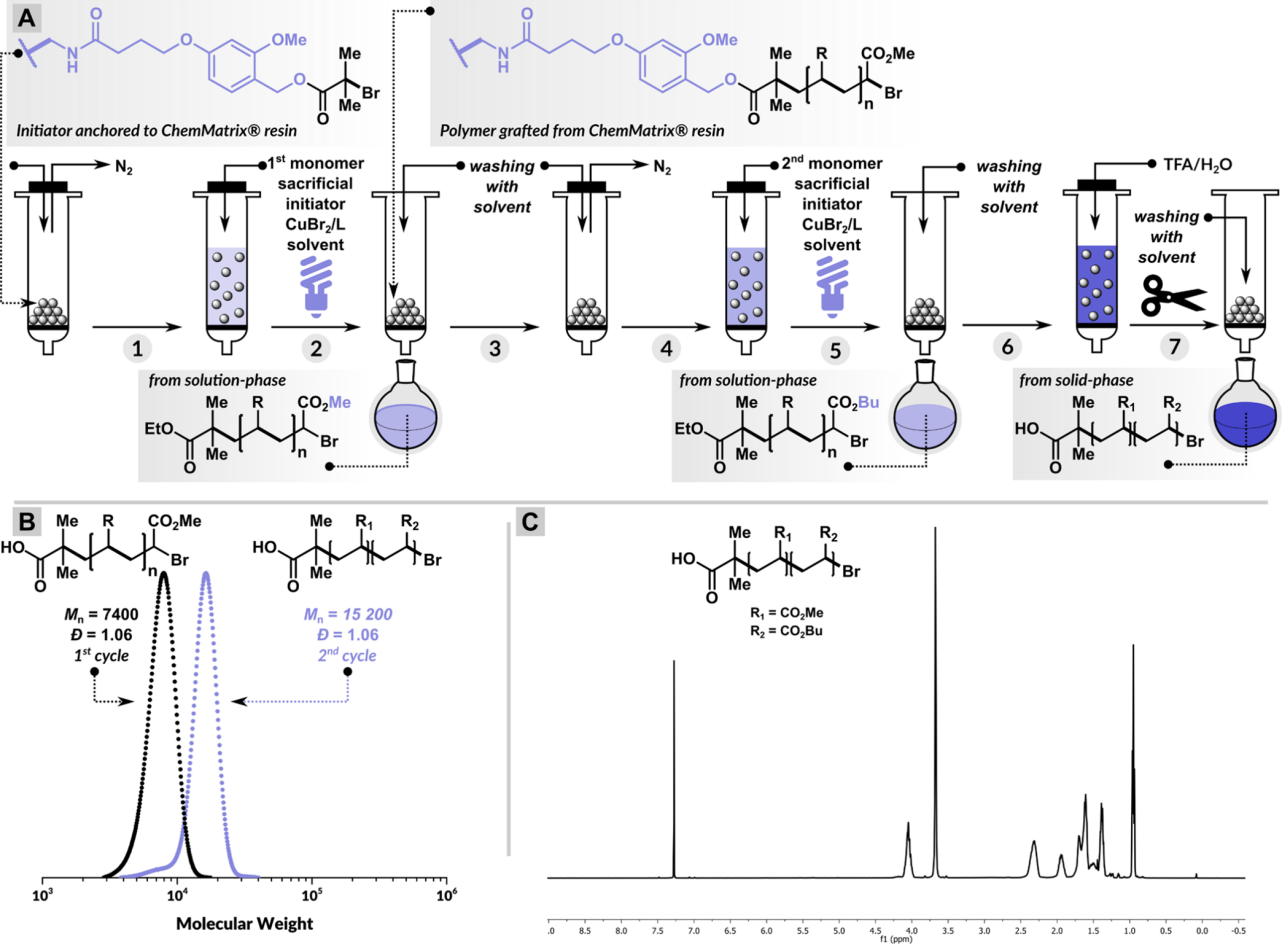
Solid-phase synthesis of diblock copolymer. (A) Synthesis protocol. (B) SEC traces of polymers cleaved after 1 and 2 cycles. (C) ^1^H NMR spectrum of poly(methyl acrylate-*b*-butyl acrylate).

After the initial success of the 1-cycle solid-phase polymer synthesis of PMA, we synthesized a block copolymer of poly(methyl acrylate-*b*-butyl acrylate) with 2 cycles (Figure 2A). During the first cycle, the polymerization of MA was carried out simultaneously in the solution phase and from CM-BiB under the same conditions as described above using the molar ratio [MA]/[EBiB]/[CuBr_2_]/[Me_6_TREN] = 200/1/0.05/0.3. After 3 h, the polymerization reaction mixture from the first cycle was separated from the heterogeneous CM and analyzed by SEC. Meanwhile, the CM resin was thoroughly washed by MeCN and without any further purification mixed with the second cycle polymerization mixture comprising butyl acrylate (BA) as the monomer, using the molar ratios [BA]/[EBiB]/[CuBr_2_]/[Me_6_TREN] = 200/1/0.05/0.3 (Figure 2A). After the second cycle, the solution phase was filtered off and analyzed by SEC (Figure 2B). The CM grafted with diblock copolymer was subjected to multiple washes followed by treatment with a TFA/H_2_O (95% v/v) mixture to release the diblock copolymer.

The SEC analysis of the solution phase revealed well-controlled polymerization in both the first cycle (Table 1, entry 1) and the second cycle (Table 1, entry 2). Furthermore, the SEC analysis of the cleaved diblock copolymer demonstrated that its molecular weight closely matched the combined molecular weights of the polymers from both cycles (Table 1, entry 3). This observation underscores the high chain-end fidelity during the solid-phase ATRP and purification processes, as also evidenced by the SEC chromatogram shifting to a higher-molecular-weight region (Figure 2). Additionally, the remarkable agreement in molecular weight and dispersity between the polymers in solution and those on the CM support highlights the swelling behavior of the CM resin, allowing the efficient diffusion of monomers and catalysts. This, in turn, confirms the suitability of the CM resin for the precise synthesis of multiblock copolymers, effectively eliminating the need for polymer purification after each block.

**Table 1:** Solid-Phase Synthesis of a Diblock Copolymer of Poly(methyl acrylate-*b*-butyl acrylate)^a^,^b^

entry	conditions	monomer	cycle	time [h]	*M*_n,th_^c^	*M*_n,app_^d^	*M*_n,abs_	*Đ*_[RI]_^e^
1	solution	MA	1	3	7700	6900	6600	1.04
2	solution	BA	2	3	10 300	9200	8200	1.05
3	solid-phase	MA + BA	1 + 2	3 + 3		15 200		1.06

a Reaction conditions: [MA] or [BA]/[EBiB]/[CuBr_2_]/[Me_6_TREN] = 200/1/0.05/0.3, [CM-BiB] = 25 mg, in MeCN (50% v/v) at ambient temperature, irradiated under UV light (370 nm, 7 mW/cm^2^), without monomer or solvent degassing. Reaction volume 5.0 mL.

b Determined by ^1^H NMR spectroscopy.

c Theoretical molecular weight (*M*_n,th_) calculated based on conversion (i.e., *M*_n,th_ = [M]_0_ × MW_[M]_ × α_[M]_ + MW_[EBiB]_).

d Apparent molecular weight (*M*_n,app_) determined by SEC in THF, based on linear PMMA calibration standards.

e Absolute molecular weight (*M*_n,abs_) calculated by universal calibration.^85^

### Varying the Target Degrees of Polymerization (DP_T_)

**Figure 3:**
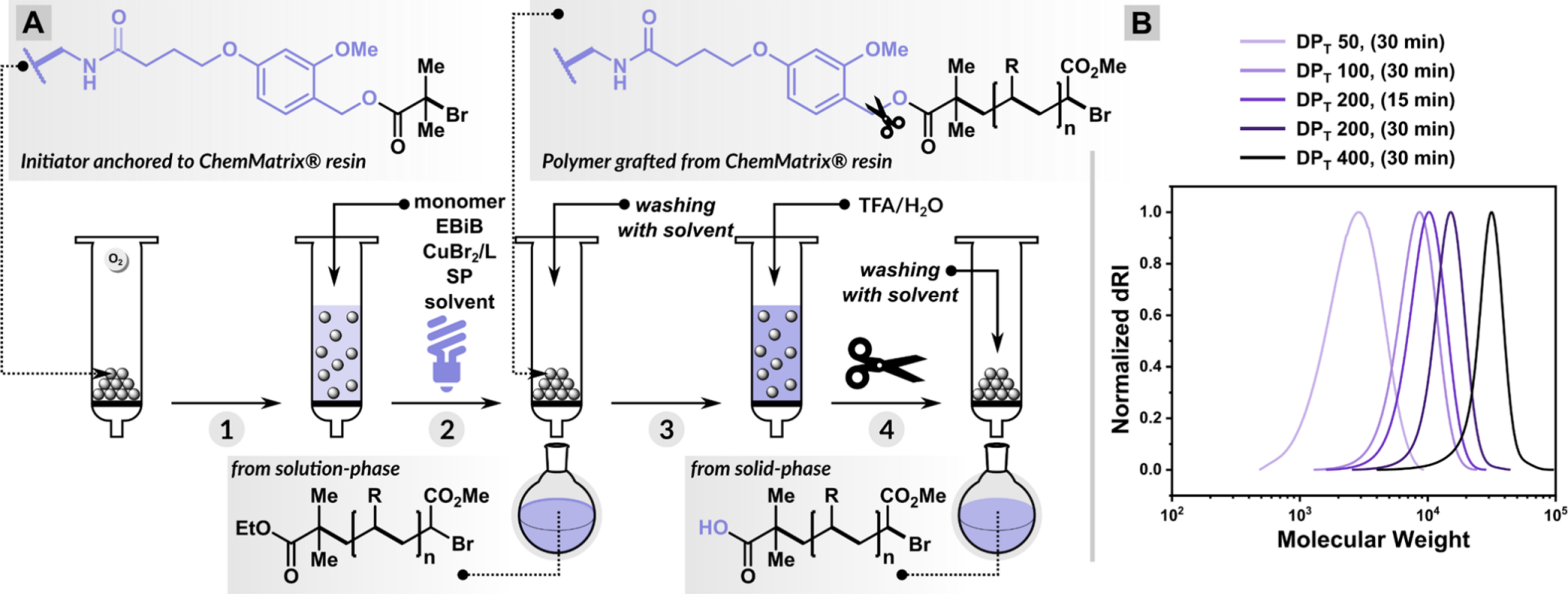
(A) Solid-phase PICAR ATRP. (B) SEC traces of polymers with varying target degrees of polymerization cleaved from the CM.

Encouraged by the efficient initiation and polymerization employing ATRP initiators immobilized on the CM support, we attempted to target various degrees of polymerizations of the polymers grafted from the CM support by varying the molar concentration of sacrificial initiators (EBiB) in the solution phase. Furthermore, considering the advantages of the fully oxygen-tolerant ATRP triggered by sodium pyruvate (SP),^84^ we decided to adopt this technique for solid-phase polymerizations to achieve rapid kinetics under benign conditions (Figure 3A). The target degrees of polymerization (DP_T_) were varied by adjusting the initiator (EBiB) concentration from 14 to 110 mM while keeping the concentration of other reagents constant (Table 2). The polymerization reactions were performed in DMSO at 50 °C by irradiation under violet LEDs (404 nm, 10 mW/cm^2^), stirring at 500 rpm in an open-to-air SPE cartridge. The results showed a high degree of control for a wide targeted DP range (50–400) (Figure 3). The monomer conversions reached 36–86% within 30 min, and the dispersity values remained very low for both polymers in the solution phase (1.09 ≤ *Đ* ≤ 1.23) as well as the cleaved polymers from the CM resin (1.10 ≤ *Đ* ≤ 1.26).

**Table 2:** Solid-Phase Synthesis of Poly(methyl acrylate) with Varying Target Degrees of Polymerization (DP_T_)^a^

entry	DP_T_	[EBiB] (mM)	time (min)	conv. (%)^b^	*M*_n,th_^c^	*M*_n,SI_^d^	*M*_n,SI,abs_^e^	*Đ*_SI_	*M*_n,CM_^d^	*M*_n,CM,abs_^e^	*Đ*_CM_
1	50	110	30	36	1500	1800	1700	1.23	2300	2200	1.26
2	100	55	30	65	5600	7300	6600	1.12	7400	6700	1.15
3	200	27.5	15	46	7900	8400	7500	1.12	8900	8000	1.13
4	200	27.5	30	74	12 700	13 600	12 000	1.09	13 600	12 000	1.10
5	400	13.8	30	86	26 600	27 600	23 500	1.13	28 200	24 000	1.10

a Reaction conditions: [MA] = 5.5 M, [EBiB] = 14–110 mM, [CuBr_2_] = 1.38 mM, [Me_6_TREN] = 4.1 mM, [SP] = 5 mM, [CM-BiB] = 20 mg, in DMSO at 50 °C, irradiated under violet LEDs (404 nm, 10 mW/cm^2^) at a stirring rate of 500 rpm in an open solid-phase extraction syringe. Reaction volume 5.0 mL.

b Monomer conversion was determined by using ^1^H NMR spectroscopy,

c Theoretical molecular weight (*M*_n,th_) calculated based on conversion (i.e., *M*_n,th_ = [M]_0_ × MW_[M]_ × α_[M]_ + MW_[EBiB]_).

d Apparent molecular weight (*M*_n,app_) determined by SEC in THF, based on linear PMMA calibration standards.

e Absolute molecular weight (*M*_n,abs_) calculated by universal calibration.^85^ Polymerizations were quenched with 1,4-bis(3-isocyanopropyl) piperazine (SnatchCat).^87^

### Expanding Monomer Scope

**Figure 4:**
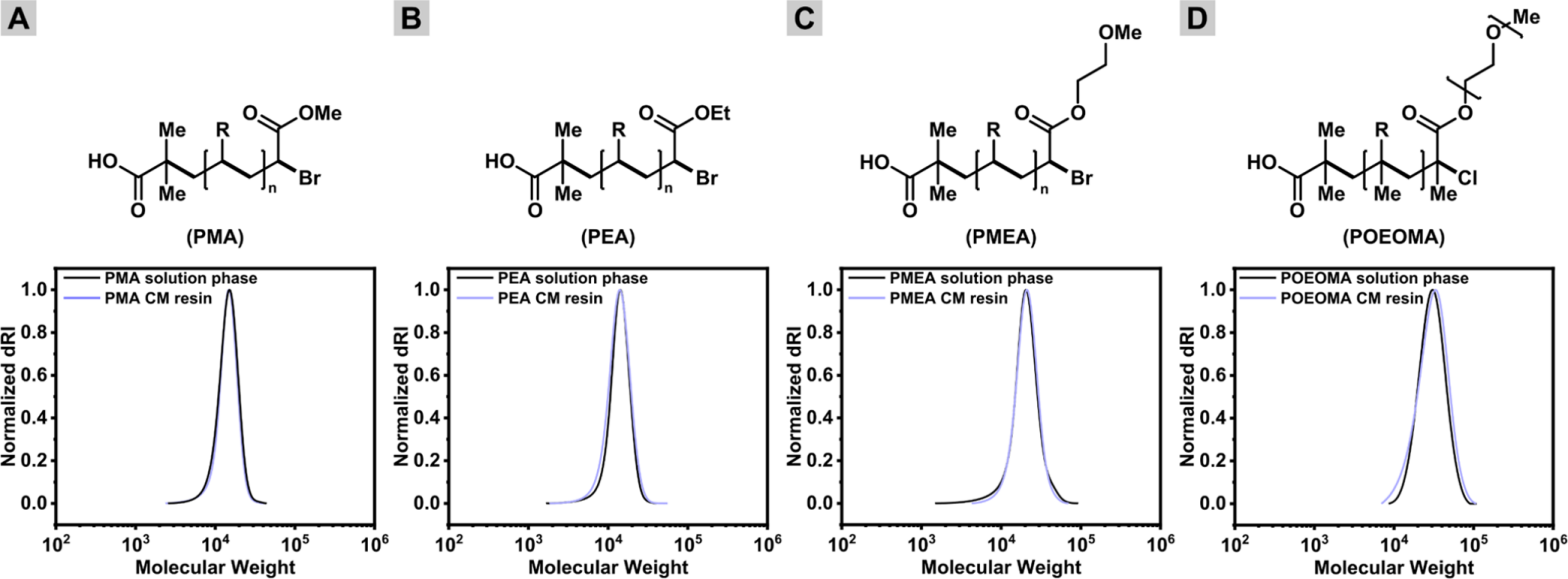
SEC traces of (A) PMA, (B) PEA, (C) PMEA, and (D) POEOMA.

The monomer scope in solid-phase PICAR ATRP was further expanded to ethyl acrylate (EA) and 2-methoxyethyl acrylate (MEA) in an organic solvent as well as for the polymerization of hydrophilic oligo(ethylene glycol) methyl ether methacrylate (OEOMA_300_) in aqueous media (Table 3). Under the optimized conditions, [M]/[EBiB]/[CuBr_2_]/[Me_6_TREN] = 200/1/0.05/0.3, all monomers reached >70% conversion within 30 min of irradiation time under violet LEDs (404 nm, 10 mW/cm^2^) (Table 3, entries 1–3). The polymerization of OEOMA_300_ was performed using [SP] = 100 mM, the CuBr_2_/TPMA complex (TPMA= tris(2-pyridylmethyl) amine) as deactivator and 2-hydroxyethyl α-bromoisobutyrate (HO-BiB) as initiator in 1× PBS (Figure S2). The molar ratios used were [OEOMA_300_]/[HO-EBiB]/[CuBr_2_]/[TPMA] = 200/1/0.2/0.6 (Table 3, entry 4). The SEC traces of polymers from both the solution phase and those grafted on the solid phase demonstrated monomodal curves with narrow molecular weight distributions (Figure 4). The results demonstrate the versatility of CM resin, which, due to the PEG-based network, showed good swelling behavior in buffered solution (1× PBS).

**Table 3:** Solid-Phase PIACR ATRP for Various Monomers^a^,^b^^,^^f^

entry	monomer	conv. (%)^f^	*M*_n,th_^c^	*M*_n,SI_^d^	*M*_n,SI,abs_^e^	*Đ*_SI_	*M*_n,CM_^d^	*M*_n,CM,abs_^e^	*Đ*_CM_
1	MA	74	12 700	13 600	12 000	1.09	13 600	11 400	1.10
2	EA	70	14 000	13 000	12 200	1.10	12 700	10 700	1.12
3	MEA	92	23 900	18 200	17 700	1.18	19 400	18 800	1.12
4^f^	OEOMA	95	28 500	23 700	27 500	1.21	27 900	33 800	1.14

a Reaction conditions: [M]/[EBiB]/[CuBr_2_]/[Me_6_TREN] = 200/1/0.05/0.1 ([MA] = 5.5 M, [EA] = 4.4 M, [MEA] = 4.4 M), [CuBr_2_] = 1.38 mM, [Me_6_TREN] = 4.1 mM, [SP] = 5 mM, [CM-BiB] = 20 mg, in DMSO at 50 °C.

b Monomer conversion was determined by using ^1^H NMR spectroscopy.

c Theoretical molecular weight (*M*_n,th_) calculated based on conversion (i.e., *M*_n,th_ = [M]_0_ × MW_[M]_ × α_[M]_ + MW_[EBiB]_).

d Apparent molecular weight (*M*_n,app_) of PMA, PEA, and PMEA was determined by SEC in THF, while POEOMA_300_ was analyzed by SEC in DMF, based on linear PMMA calibration standards.

e Absolute molecular weight (*M*_n,abs_) calculated by universal calibration.^85^ Polymerizations were quenched with 1,4-bis(3-isocyanopropyl) piperazine (SnatchCat).^87^

f Reaction conditions = [OEOMA_300_]/[HO-EBiB]/[CuBr_2_]/[TPMA] = 200/1/0.2/0.6, [OEOMA_300_] = 300 mM, [HO-EBiB] = 1.5 mM, [CuBr_2_] = 0.3, [TPMA] = 0.9 mM, [SP] = 100 mM, [CM-BiB] = 20 mg, in 1× PBS at r.t. Reaction volume 5.0 mL. Irradiated under violet LEDs (404 nm, 10 mW/cm^2^) for 30 min at a stirring rate of 500 rpm in an open solid-phase extraction syringe.

### Solid-Phase PICAR ATRP for the Synthesis of Multiblock Copolymers

**Figure 5:**
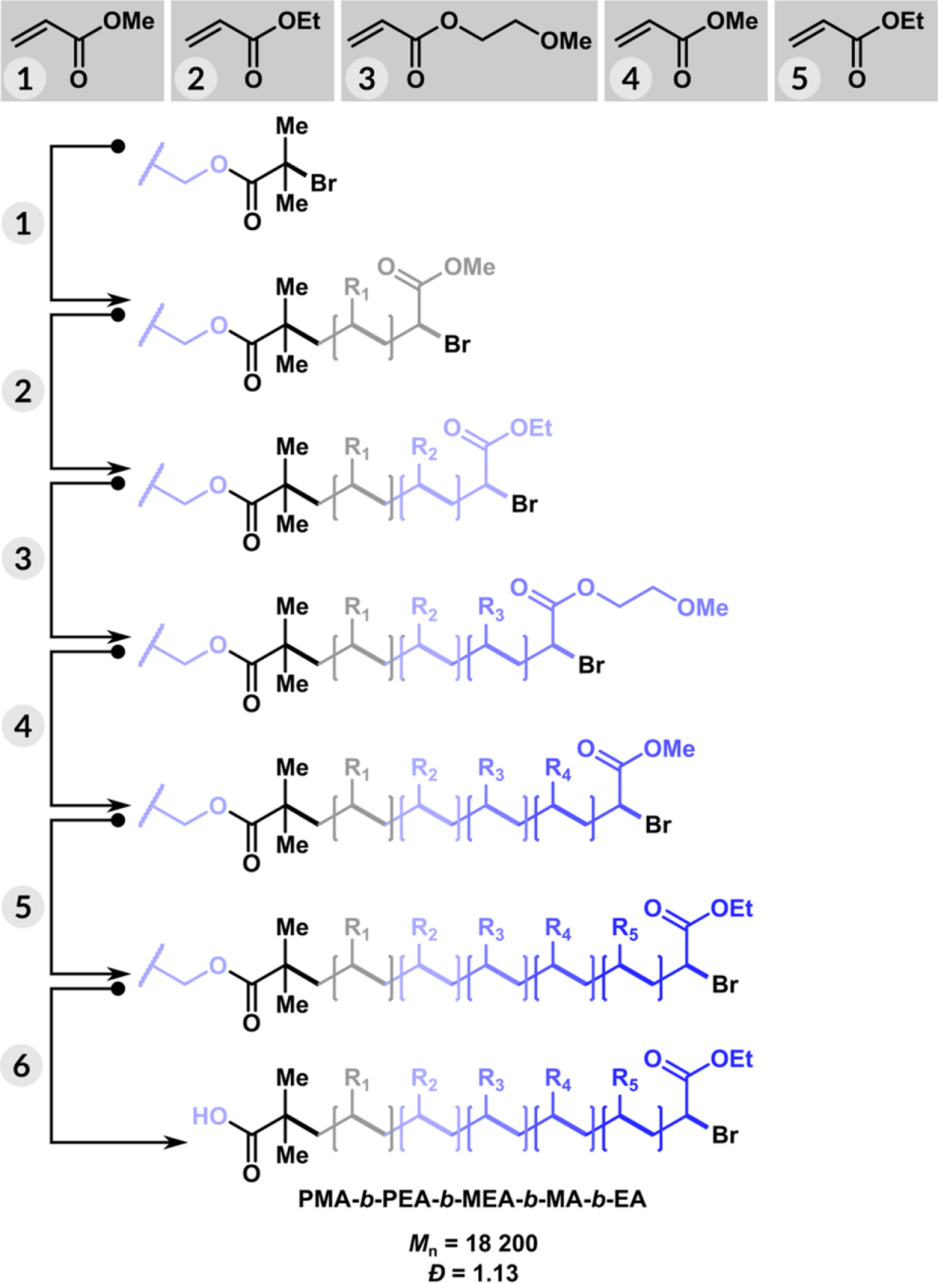
Solid-phase synthesis of PMA-*b*-PEA-*b*-PMEA-*b*-PMA-*b*-PEA penta-block copolymer. Reaction conditions: [M]/[EBiB]/[CuBr_2_]/[Me_6_TREN] = 100/1/0.1/0.2, [M] = 5.5 M, [EBiB] = 55 mM, [CuBr_2_] = 1.38 mM, [Me_6_TREN] = 4.1 mM, [SP] = 5 mM, [CM-BiB] = 20 mg, in DMSO at 50 °C, irradiated for 10 min under violet LEDs (404 nm, 10 mW/cm^2^) at a stirring rate of 500 rpm in an open solid-phase extraction syringe. Reaction volume 5.0 mL. Polymerizations were quenched with 1,4-bis(3-isocyanopropyl) piperazine (SnatchCat).^87^ (1) [M] = [MA], (2) [M] = [ME], (3) [M] = [MEA], (4) [M] = [MA], (5) [M] = [EA], (6) TFA/H_2_O (95% v/v).

The solid-phase PICAR-ATRP for synthesis of homopolymer of PMA, PMA-*b*-PEA diblock, PMA-*b*-PEA-*b*-PMEA triblock, PMA-*b*-PEA-*b*-PMEA-*b*-PMA tetrablock and PMA-*b*-PEA-*b*-PMEA-*b*-PMA-*b*-PEA penta-block copolymers were successfully carried out under conditions described above (Figure 5). Each successive polymerization cycle was continued by simply filtering the polymerized solution from the previous block and introducing the next polymerization mixture with the desired monomer (Supporting Information). The final molecular weight of cleaved polymers showed good agreement with the theoretical molecular weights and displayed low dispersity values (Tables S1–S5). The SEC analysis of cleaved block copolymers from the solid phase showed monomodal peaks with a narrow molecular weight distribution and precise control over molecular weight (Figure S3).

Next, multiblock copolymer synthesis in solution was compared to the solid-phase approach. During the solution-phase synthesis, each polymer obtained had to be reprecipitated twice in a water/methanol mixture, dried, dissolved in dichloromethane, dried, passed through neutral alumina, and reprecipitated in water/methanol or hexane (Supporting Information). In comparison, the solid phase was simply washed with solvents to remove copper residues and unreacted monomers for purification. The first block was straightforwardly synthesized by PICAR ATRP of MA in DMSO phase using EBiB as the initiator. The resulting PMA was then used as a precursor for chain extension. Gradually, the solubility of the polymer decreased due to the increase in molecular weight, which prevented the use of the same stoichiometric ratio for the synthesis of the next block. The results showed that after the formation of the triblock copolymer, a high stoichiometric ratio of the monomer was required and the resulting polymer had a higher dispersity (*Đ* ∼ 1.7) (Figure S10). These results further demonstrated the advantages of the solid-phase approach, as the monomer/initiator ratio could be kept constant, and the DP_T_ was not limited by physicochemical constraints. In addition, a penta-block copolymer could be obtained in less than 2 h using the solid-phase method, a significant improvement over the several days required for the solution-phase method.

### Investigating the Limitation in the Molecular Weight of Immobilized Polymer

The reported mesh size of the cross-linked CM support is 35–100 mesh. The largest molecular weight of the grafted polymer chain from the solid support can be limited due to the confinement of the living polymer chains within the cross-linked CM structure. To evaluate the highest target degree of polymerization that can be achieved from the CM-BiB support, the cycling efficiency of the polymerization on the solid support was investigated.

**Figure 6:**
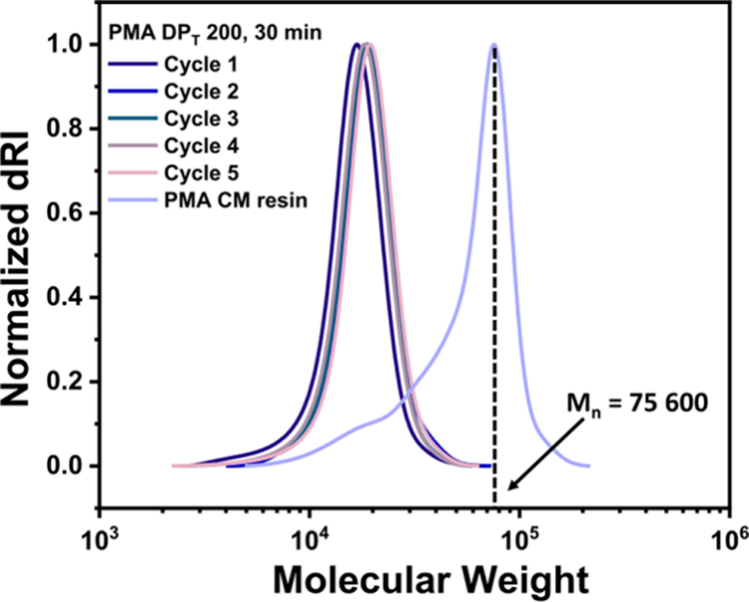
SEC traces of polymers in Table S6, entries 1–6.

The ATRP initiator-functionalized CM-BiB was used for five successive cycles of solid-phase polymerization of (MA), using the optimized molar ratios [MA]/[EBiB]/[CuBr_2_]/[Me_6_TREN] = 200/1/0.05/0.3. The solution phase after each consecutive polymerization was analyzed by ^1^H NMR and SEC revealing high conversion (>80%) and excellent control over molecular weight and dispersity of the polymers was achieved (Figure 6 and Table S6). After the fifth cycle, the CM resin immobilized with polymer was washed thoroughly and subjected to acid cleavage with a TFA/H_2_O (95% v/v) mixture to a polymer. The SEC analysis of the cleaved polymer backbone exhibited higher apparent molecular weight than the polymers synthesized during each cycle, as well as bimodality and lower-than-expected total apparent molecular weight.

To determine the ratio of the initiated polymer chains and noninitiated polymer chains within the resin during each successive cycle, the weight-based GPC chromatogram (Figure 6) was converted to a number-based GPC trace (Figure S3A). The number-based GPC peak was deconvoluted to five Gaussian-distributed peaks corresponding to cumulative MW from each cycle, with an *R*^2^ value of ca. 99% (Figure S3B). It revealed the relative areas under low-MW and high-MW peaks were approximately 8 and 92%, respectively. This observation confirms the heterogeneous distribution of functional groups in the CM network. The nonuniformity of pores in the CM resin restricts the continued growth of some of the living polymer chains (≈10%), whereas the remaining polymer chains (≈90%) potentially continue to grow as they remain accessible to monomer and catalyst throughout the consecutive cycles of polymerization and yield final polymers of desired composition with high chain-end fidelity.

## Conclusions

In summary, we combine the highly efficient PICAR ATRP method with the advantages of solid-phase synthesis for the synthesis of multiblock copolymers without the need for cumbersome and wasteful purification steps between each block synthesis. Moreover, employing fully oxygen-tolerant photoinduced ATRP not only eliminated the requirement for deoxygenation steps but also exhibited rapid kinetics, maintaining excellent control over the molecular weight and dispersity of the synthesized polymers. We found the ChemMatrix resin to be critical. Its excellent swelling properties in various solvents allowed the copper complexes to access radicals and dormant polymers anchored to the solid support, resulting in excellent control over the polymerization process. In addition, we compared PICAR ATRP in solution to the solid-phase approach. The solution-based synthesis of a tetrablock copolymer resulted in a polymer with high dispersity (*Đ* ∼ 1.7), possibly due to limited solubility. In contrast, the solid-phase method provided a penta-block copolymer with low dispersity (*Đ* = 1.13) in less than 2 h. This approach, combined with automation and machine learning, has the potential to become a high-throughput platform for creating polymers with diverse architectures and advanced polymer hybrids.

## Supporting Information Available

Details of experimental procedures and additional experiments and polymerization results (PDF)
