# Supplementary material for: Solid-Phase Synthesis of Well-Defined Multiblock Copolymers by Atom Transfer Radical Polymerization
Source: J Am Chem Soc. 2024 Jul 30;146(32):22247–56. doi: 10.1021/jacs.4c03675 (PMC11328128; doi:10.1021/jacs.4c03675)
Supplement: Supplementary file 1 — ja4c03675_si_001.pdf [file ja4c03675_si_001.pdf]

Solid-Phase Synthesis of Well-Defined Multiblock Copolymers by Atom Transfer Radical Polymerization

Grzegorz Szczepaniak[†,1,2] Kriti Kapil[†],[1] Samuel Adida,[1] Gorkem Yilmaz,[1] Khidong Kim,[1] Ting-Chih Lin,[1] Hironobu Murata,[1] Krzysztof Matyjaszewski[1]*

[1] Department of Chemistry, Carnegie Mellon University, 4400 Fifth Avenue, Pittsburgh, PA 15213, United States.

[2] Faculty of Chemistry, University of Warsaw, Pasteura 1, 02-093 Warsaw, Poland.

[†] These authors have contributed equally to the manuscript.

Table of Contents

Experimental Details 3
Materials 3
Instrumentation 3
Nuclear Magnetic Resonance (NMR) 3
Size Exclusion Chromatography (SEC) 3
Synthesis of ATRP-initiator functionalized ChemMatrix resin (CM-BIB) 4
Digital images for reaction set-up of solid-phase polymer synthesis by PICAR ATRP 5
Polymerization results of multiblock copolymer synthesis 5
Polymerization in aqueous medium: solid-phase synthesis of POEOMA 9
Deconvolution of SEC trace in Figure 5 10
^{1}H NMR spectrum of poly(methyl acrylate-b-butyl acrylate) 12
Full SEC traces 12
Synthesis of block copolymers by PICAR ATRP in the solution phase (DMSO) 15

Experimental Details

Materials

All chemicals were purchased from commercial sources and used as received unless otherwise noted. Tris(2-pyridylmethyl) amine (TPMA, 99%), tris[2-(dimethylamino) ethyl] amine (Me_6TREN, 99%) were purchased from *AmBeed*. Copper (II) bromide ($CuBr_2$, 99.99%), 2-hydroxyethyl 2-bromoisobutyrate (HO-EBIB ,95%), ethyl α-bromoisobutyrate (EBIB, 98%), 1,4-bis(3-isocyanopropyl) piperazine (QA), triethanolamine (TEOA, ≥99.0%), 4-(4-hydroxymethyl-3-methoxyphenoxy)butyric acid, polymer-bound to ChemMatrix® resin (0.40–0.65 mmol/g -OH, CM-HMPB), trifluoroacetic acid (TFA, 99%) 1,4-bis(3-isocyanopropyl) piperazine (QA, ≥95%), triethanolamine (TEOA, ≥99.0%), were purchased from *Sigma-Aldrich*. 10X PBS was purchased from *Thermo Fisher Scientific*. Methyl acrylate (MA), butyl Acrylate (BA) ethyl acrylate (EA), 2-methoxyethyl acrylate (MEA), oligo(ethylene glycol) methyl ether methacrylate (average M_n = 300, $OEOMA_{300}$) was purchased from *Sigma-Aldrich* and passed through a column of basic alumina to remove inhibitor prior to use. Water (HPLC grade), Acetonitrile (HPLC grade) and dimethyl sulfoxide (DMSO, ≥99.7%) were purchased from *Fisher.* Sodium pyruvate (SP, >97.0%) was purchased from *TCI*. Fritted flangeless polypropylene reservoir; 1 Frit, 20 Micron (RFT1FR8P) were purchased from *United Chem* (UCT). The violet LEDs were purchased from aspectLED.

Instrumentation

Nuclear Magnetic Resonance (NMR)

^1H NMR spectra were recorded on *Bruker* Avance III 500 MHz spectrometers with D_2O, $CDCl_3$ or DMSO-d^6 used as the solvent.

Size Exclusion Chromatography (SEC)

SEC measurements of poly(methyl acrylate), poly(ethyl acrylate), poly(methoxyethyl acrylate) and multi-block copolymers were conducted using *PSS* columns (SDV 10^2, 10^3, 10^5 Å) with THF as an eluent at 35 °C and the flow rate of 1 mL/min. Linear poly(methyl methacrylate) standards were used for calibration.

SEC measurements of p(OEOMA300) were performed using *PSS* columns (GRAM 10^2 , 10^3 , 10^5 Å) with DMF containing 0.05 M LiBr as an eluent at 50 °C and a 1 mL/min flow rate. Linear poly(methyl methacrylate) standards were used for calibration.

Synthesis of ATRP-initiator functionalized ChemMatrix resin (CM-BIB)

ChemMatrix resin bearing benzylic hydroxyl groups (0.40-0.65 mmol/g, 1 g) was taken in a 100 mL round bottom flask with a magnetic stirrer. DCM (50 mL) and triethyl amine (0.8 mmol, 112 μL) were taken into the flask and placed in an ice bath. In the reaction flask, α-bromoisobutyryl bromide (BIB-Br, 0.8 mmol, 100 μL) in DCM (10 mL) was slowly added. The reaction mixture was kept stirring overnight at room temperature and filtered off. The filtered solid was washed with water, methanol, and diethyl ether and dried under vacuum to give pure CM-BiB (Figure 1).

Fourier Transform Infrared Spectroscopy (FTIR) analysis of native CM (CM-OH) and ATRP initiator functionalized CM (CM-BiB) revealed diminishing - OH peak (broad peak at 3300 cm^{-1}) and a sharp peak for ester functional group (OC = O at 1700 cm^{-1}) that confirm the functionalization. Additionally, other peaks remained unchanged showing that the reaction conditions did not degrade the resins (Figure S1).

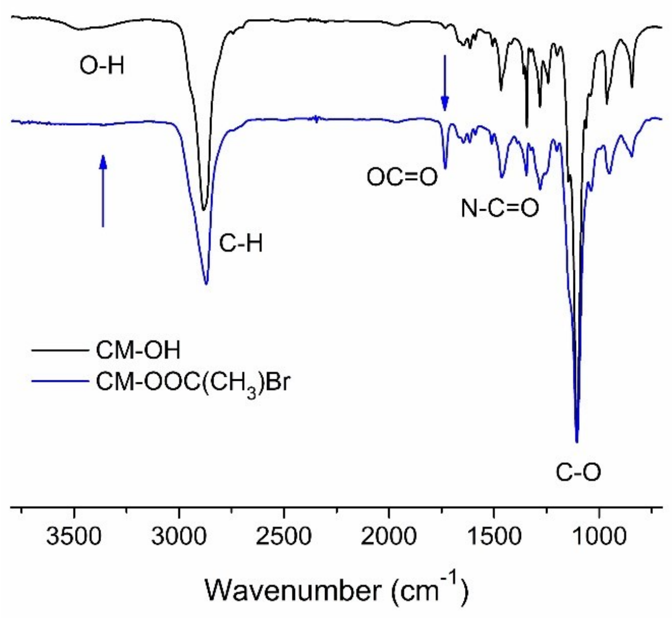

Figure S1. Fourier Transform Infrared Spectroscopy (FTIR) of CM-OH and CM-BiB.

Two separate samples of ChemMatrix resin functionalized with ATRP initiator (CM-BiB) were subjected to elemental analysis. The analysis revealed the following average elemental composition (wt%): C - 55.34, H - 8.46, N - 1.05, Br - 0.75. The initiator loading was calculated to be 0.09 mmol/g based on the elemental composition.

Digital images for reaction set-up of solid-phase polymer synthesis by PICAR ATRP

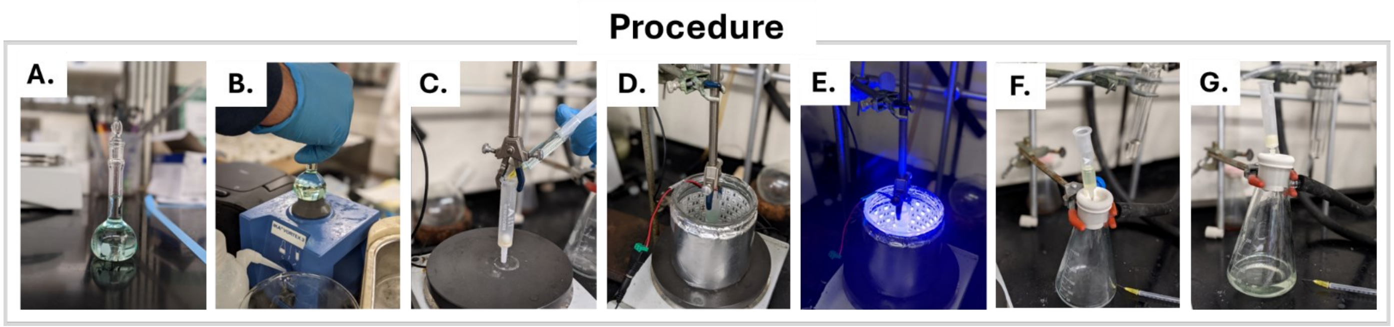

Figure S2. A-G represents stepwise procedure for solid-phase polymer synthesis by PICAR ATRP.

Polymerization results of multiblock copolymer synthesis

Table S1. Solid-Phase Synthesis of PMA[a]

Cycle	Monomer	Conv. (%)	$M_{n,th}$	$M_{n,app}$	$M_{n,abs}$	$Đ$	CM (mg)
1	MA	48	4100	4100	3600	1.16	-
CM		-	4100	4500	4000	1.22	42

[a]Reaction conditions: [MA]/[EBiB]/[$CuBr_2$]/[Me_6TREN] = 100/1/0.1/0.2, [MA] = 5.5 M, [EBiB] = 55 mM, [$CuBr_2$] = 1.38 mM, [Me_6TREN] = 4.1 mM, [SP] = 5 mM, [CM-BiB] = 20 mg, in DMSO at 50 °C, irradiated for 10 min under violet LEDs (λ = 404 nm, 10 mW/cm^2) at a stirring rate of 500 rpm in an open reaction vessel. Reaction volume 5.0 mL. [b]Monomer conversion was determined by using ^{1}H NMR spectroscopy. All measurements were analyzed using GPC (THF as eluent) calibrated to poly(methyl methacrylate) standards. Polymerizations were quenched with 1,4-bis(3-isocyanopropyl)piperazine (SnatchCat).

Table S2. Solid-Phase Synthesis of PMA-*b*-PEA[a]

Cycle	Monomer	Conv. (%)	$M_{n,th}$	$M_{n,app}$	$M_{n,abs}$	*Đ*	CM (mg)
1	MA	46	4000	4500	4000	1.18	-
2	EA	43	4300	4800	4200	1.19	-
CM		-	8300	9200	-	1.17	68

[a]Reaction conditions: [M]/[EBiB]/[$CuBr_2$]/[Me_6TREN] = 100/1/0.1/0.2, , M = [MA]/[EA] = 5.5 M, [EBiB] = 55 mM, [$CuBr_2$] = 1.38 mM, [Me_6TREN] = 4.1 mM, [SP] = 5 mM, [CM-BiB] = 20 mg, in DMSO at 50 °C, irradiated for 10 min under violet LEDs (λ = 404 nm, 10 mW/cm^2) at a stirring rate of 500 rpm in an open reaction vessel. Reaction volume 5.0 mL. [b]Monomer conversion was determined by using ^{1}H NMR spectroscopy. All measurements were analyzed using GPC (THF as eluent) calibrated to poly(methyl methacrylate) standards. Polymerizations were quenched with 1,4-bis(3-isocyanopropyl)piperazine (SnatchCat).

Table S3. Solid-Phase Synthesis of PMA-*b*-PEA-*b*-MEA[a]

Cycle	Monomer	Conv. (%)	$M_{n,th}$	$M_{n,app}$	$M_{n.abs}$	*Đ*	CM (mg)
1	MA	55	4700	4500	4000	1.15	-
2	EA	42	4200	5100	4500	1.19	-
3	MEA	74	9600	9700	8200	1.16	-
CM		-	18 500	15 700	-	1.17	108

[a]Reaction conditions: [M]/[EBiB]/[$CuBr_2$]/[Me_6TREN] = 100/1/0.1/0.2, M = [MA]/[EA]/[MEA] = 5.5 M, [EBiB] = 55 mM, [$CuBr_2$] = 1.38 mM, [Me_6TREN] = 4.1 mM, [SP] = 5 mM, [CM-BiB] = 20 mg, in DMSO at 50 °C, irradiated for 10 min under violet LEDs (λ = 404 nm, 10 mW/cm^2) at a stirring rate of 500 rpm in an open reaction vessel. Reaction volume 5.0 mL. [b]Monomer conversion was determined by using ^{1}H NMR spectroscopy. All measurements were analyzed using GPC (THF as eluent) calibrated to poly(methyl methacrylate) standards. Polymerizations were quenched with 1,4-bis(3-isocyanopropyl)piperazine (SnatchCat).

Table S4. Solid-Phase Synthesis of PMA-*b*-PEA-*b*-MEA-*b*-MA[a]

Cycle	Monomer	Conv. (%)	$M_{n,th}$	$M_{n,app}$	$M_{n,abs}$	*Đ*	CM (mg)
1	MA	36	3100	2900	2600	1.27	-
2	EA	24	2400	3000	2700	1.21	-
3	MEA	60	7800	8500	7300	1.15	-
4	MA	43	3700	4500	4000	1.16	-
CM		-	17 000	16 700	-	1.13	177

[a]Reaction conditions: [M]/[EBiB]/[$CuBr_2$]/[Me_6TREN] = 100/1/0.1/0.2, M = [MA]/[EA]/[MEA] = 5.5 M, [EBiB] = 55 mM, [$CuBr_2$] = 1.38 mM, [Me_6TREN] = 4.1 mM, [SP] = 5 mM, [CM-BiB] = 20 mg, in DMSO at 50 °C, irradiated for 10 min under violet LEDs (λ = 404 nm, 10 mW/cm^2) at a stirring rate of 500 rpm in an open reaction vessel. Reaction volume 5.0 mL. [b]Monomer conversion was determined by using ^{1}H NMR spectroscopy. All measurements were analyzed using GPC (THF as eluent) calibrated to poly(methyl methacrylate) standards. Polymerizations were quenched with 1,4-bis(3-isocyanopropyl)piperazine (SnatchCat).

Table S5. Solid-Phase Synthesis of PMA-*b*-PEA-*b*-MEA-*b*-MA-*b*-EA[a]

Cycle	Monomer	Conv. (%)	$M_{n,th}$	$M_{n,app}$	$M_{n,abs}$	*Đ*	CM (mg)
1	MA	26	2200	3100	2800	1.20	-
2	EA	26	2600	2900	2600	1.22	-
3	MEA	59	7700	8300	7000	1.15	-
4	MA	40	3400	4200	3200	1.16	-
5	EA	37	3700	4700	3300	1.17	-
CM		-	19 600	18 200	-	1.13	192

[a]Reaction conditions: [M]/[EBiB]/[$CuBr_2$]/[Me_6TREN] = 100/1/0.1/0.2, M = [MA]/[EA]/[MEA] =5.5 M, [EBiB] = 55 mM, [$CuBr_2$] = 1.38 mM, [Me_6TREN] = 4.1 mM, [SP] = 5 mM, [CM-BiB] = 20 mg, in DMSO at 50 °C, irradiated for 10 min under violet LEDs (λ = 404 nm, 10 mW/cm^2) at a stirring rate of 500

rpm in an open reaction vessel. Reaction volume 5.0 mL. [b]Monomer conversion was determined by using ^{1}H NMR spectroscopy. All measurements were analyzed using GPC (THF as eluent) calibrated to poly(methyl methacrylate) standards. Polymerizations were quenched with 1,4-bis(3-isocyanopropyl)piperazine (SnatchCat).

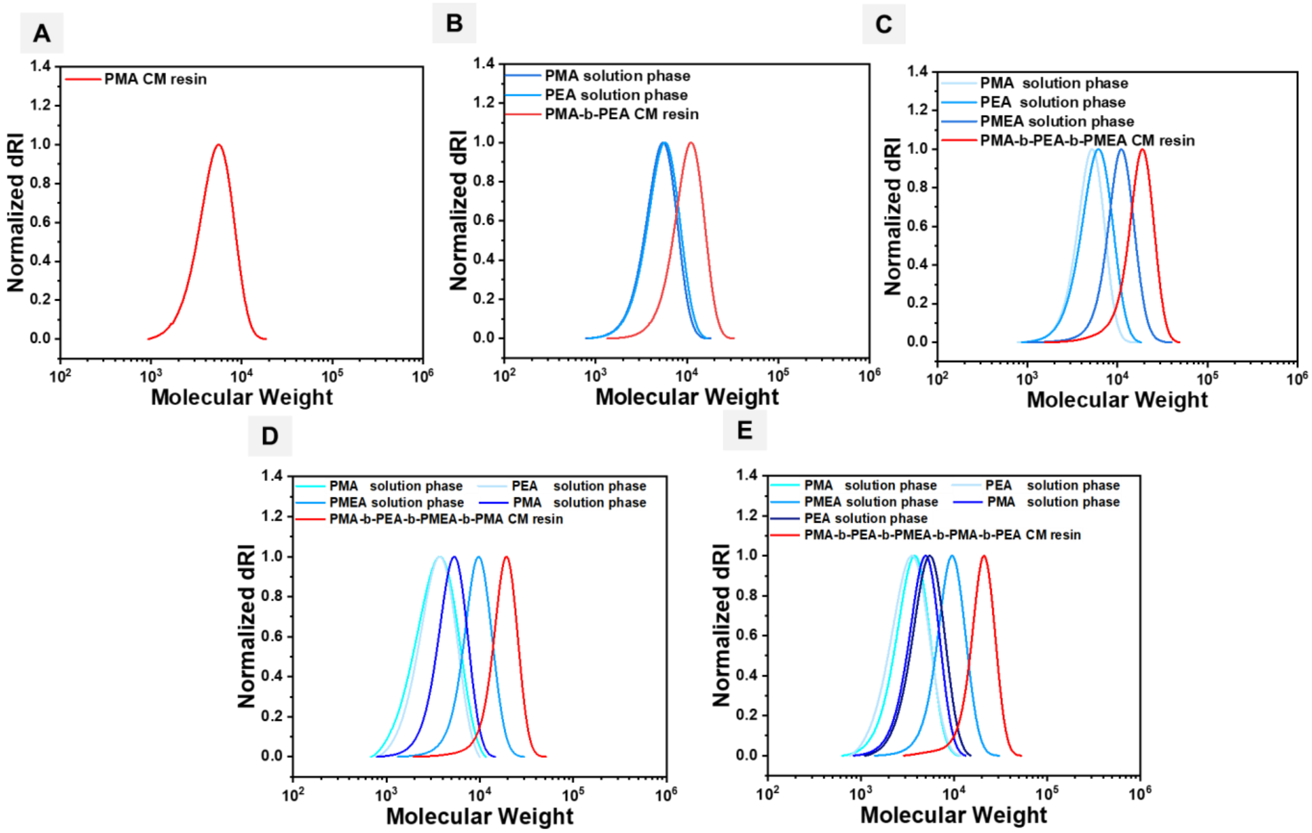

Figure S3. SEC traces of (A) homopolymer of PMA, (B) diblock copolymer of PMA-*b*-PEA, (C) triblock copolymer comprising of PMA-*b*-PEA-*b*-PMEA,(D) tetrablock copolymer of PMA-*b*-PEA-*b*-PMEA-*b*-PMA, and (E) pentablock copolymer of PMA-*b*-PEA-*b*-PMEA-*b*-PMA-*b*-PEA.

Polymerization in aqueous medium: solid-phase synthesis of POEOMA

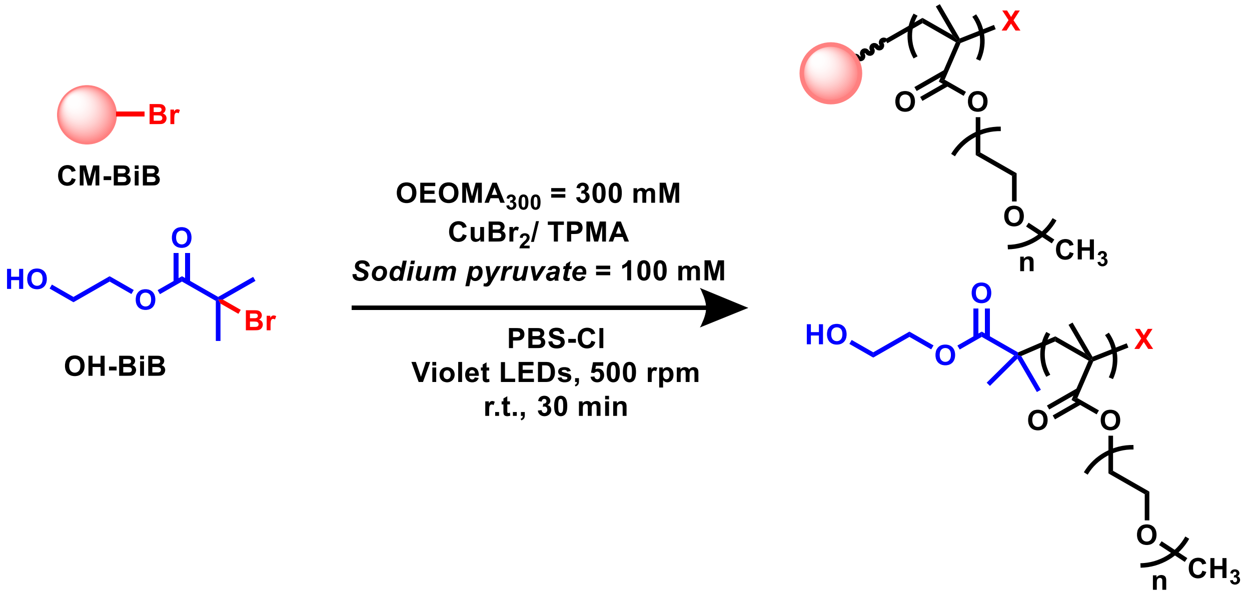

Figure S4: Schematic representation of solid-phase PICAR ATRP for synthesis of $POEOMA_{300}$.

Prior to polymerization, stock solutions of HOBiB (15.8 mg in 1.0 mL DMSO), $CuBr_2$ (33.5 mg in 20.0 mL DMSO), TPMA (13.1 mg in 1.0 mL DMSO) and SP (110 mg in 2 mL HPLC water) were prepared.

In a 5 mL volumetric flask, 450 mg of $OEOMA_{300}$ was weighed. $CuBr_2$ stock (200 µL), TPMA stock (100 µL), HO-EBiB stock (100 µL), SP stock (1 mL), DMSO (100 µL) and 10X PBS solution (500 µL) were then added. Finally, water was added to the mark on the volumetric flask, and the reaction mixture was stirred on a vortex (Figure S1D). The final concentrations were $OEOMA_{300}$ (300 mM), HOBiB (1.5 mM), SP (100 mM), $CuBr_2$ (0.3 mM), TPMA (0.9 mM), DMSO (10% v/v). In an 8 mL SPE cartridge fitted with a 0.2 microns filter, 20 mg of CM-BIB was transferred, followed by 4.4 mL of the ATRP cocktail along with a magnetic stirrer. The polymerization mixture was stirred at 500 rpm for 30 min for CM-BIB to swell and homogenize before irradiating the cartridge under violet LEDs (λ = 404 nm, 10 mW/cm^2) at a stirring rate of 500 rpm fully open to air. Samples were taken and analyzed by ^{1}H NMR and SEC techniques.

Deconvolution of SEC trace in Figure 5

To determine the ratio of the initiated polymer chains and non-initiated polymer chains, weight-based GPC chromatogram was converted to number-based GPC trace via dividing refractive index signal (RI) at each point by molecular weight (MW). Normalized RI/MW value plotted as black line in (Figure S3A) indicated the relative number of chains at each MW.

Number-based bimodal GPC peak was deconvoluted to five Gaussian-distributed peaks with high accuracy of R^2 value ca. 99%. (Figure S3B). The area under the predominant high MW peak was approximately 85%. This high MW peak was associated with the block copolymer chain at a specific molecular weight (59 200). The summation of areas under low MW peaks and a slightly higher MW peak was approximately 15%. The ratio of the areas under the curve indicate that the initiation efficiency of the macroinitiator was ~ 90%.

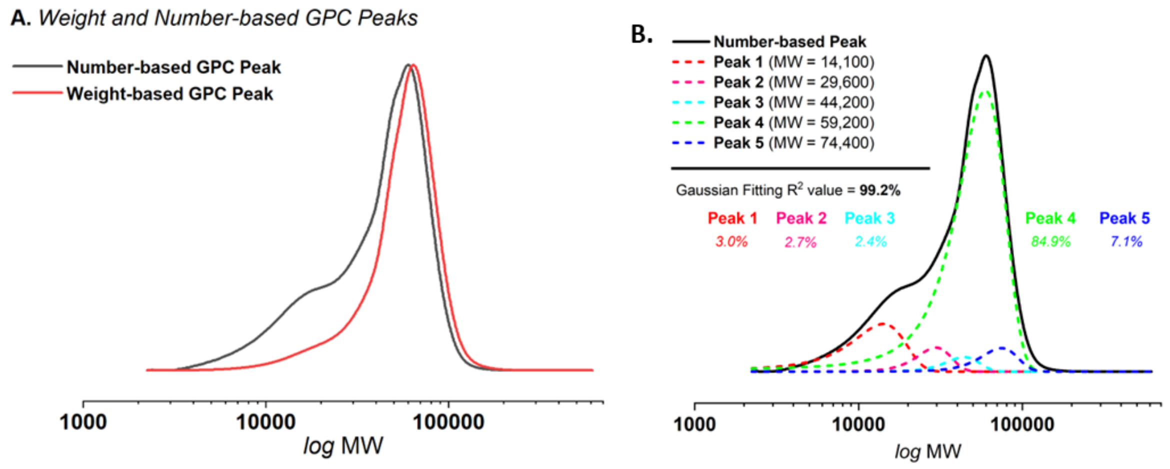

Figure S5. (A) Weight-based GPC chromatogram converted to number-based GPC trace. Deconvolution of number-based bimodal GPC peak to (B) five Gaussian-distributed peaks with high accuracy of R^2 value ca. 99%.

Table S6. Solid-Phase PICAR ATRP of MA (DP_T = 200, 30 min)[a]

Cycle	[b]Conv. (%)	[c]$M_{n,th}$	[d]$M_{n,app}$	[e]$M_{n,abs}$	*Đ*	CM (mg)
1	82	14 100	14 900	14 000	1.14	-
2	90	15 500	17 600	16 000	1.11	-
3	85	14 600	16 900	15 600	1.12	-
4	87	15 000	16 700	15 400	1.12	-
5	88	15 200	17 600	16 200	1.12	-
CM	-	74 400	45 800	49 000	1.37	119

[a]Reaction conditions: [MA]/[EBiB]/[$CuBr_2$]/[Me_6TREN] = 200/1/0.05/0.1, [MA] = 5.5 M, [EBiB] = 27.5 mM, [$CuBr_2$] = 1.38 mM, [Me_6TREN] = 4.1 mM, [SP] = 5 mM, [CM-BiB] = 20 mg, in DMSO at 50 °C. irradiated for 30 min under violet LEDs (λ = 404 nm, 10 mW/cm^2) at a stir rate of 500 rpm in an open reaction vessel. Reaction volume 5.0 mL. [b]Monomer conversion was determined by using ^{1}H NMR spectroscopy. [c]Theoretical molecular weight ($M_{n,Th}$) Calculated based on conversion (i.e., $M_{n,th}$ = $[M]_0$ × $MW_{[M]}$ × α[M] × $MW_{[EbiB]}$, [d] Apparent molecular weight ($M_{n,app}$) determined by SEC in THF, based on linear PMMA calibration standards, [e]Absolute molecular weight ($M_{n,abs}$) calculated by MHS correction using K & a parameters.[86] Polymerizations were quenched with 1,4-bis(3-isocyanopropyl) piperazine (SnatchCat).

^{1}H NMR spectrum of poly(methyl acrylate-b-butyl acrylate)

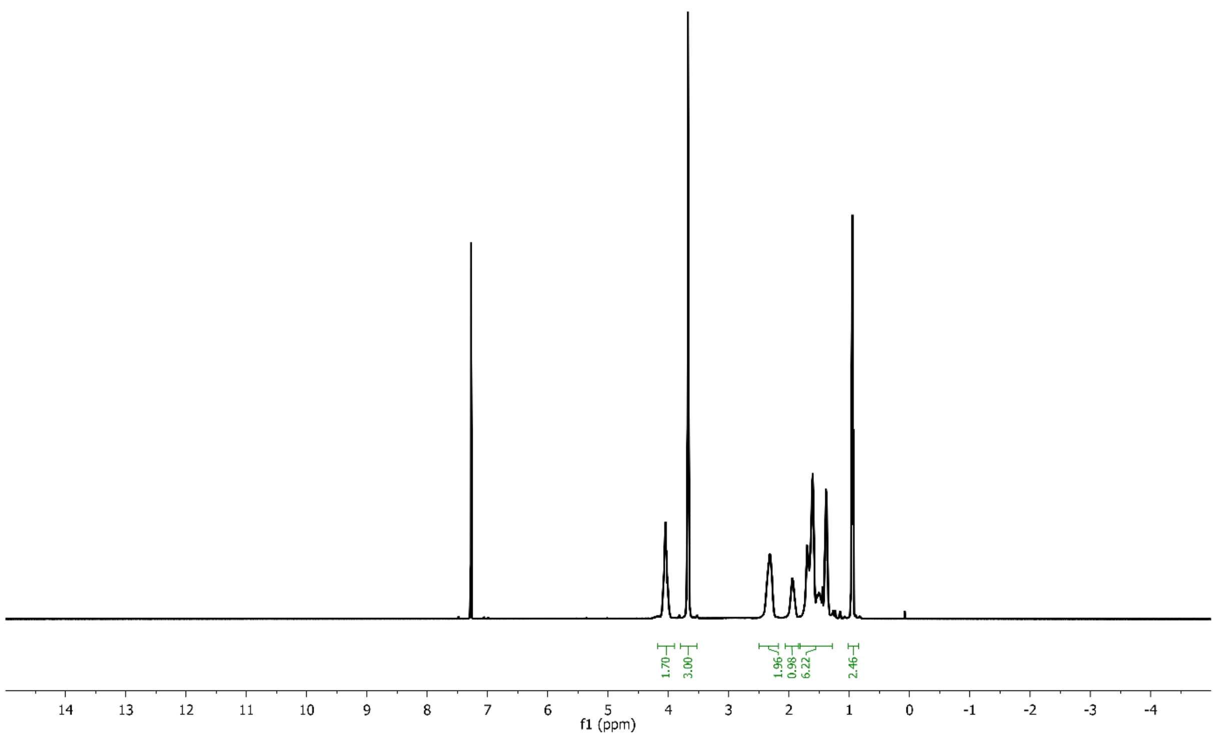

Full SEC traces

The raw data obtained from the SEC analysis has been plotted below, representing the results included in the main paper.

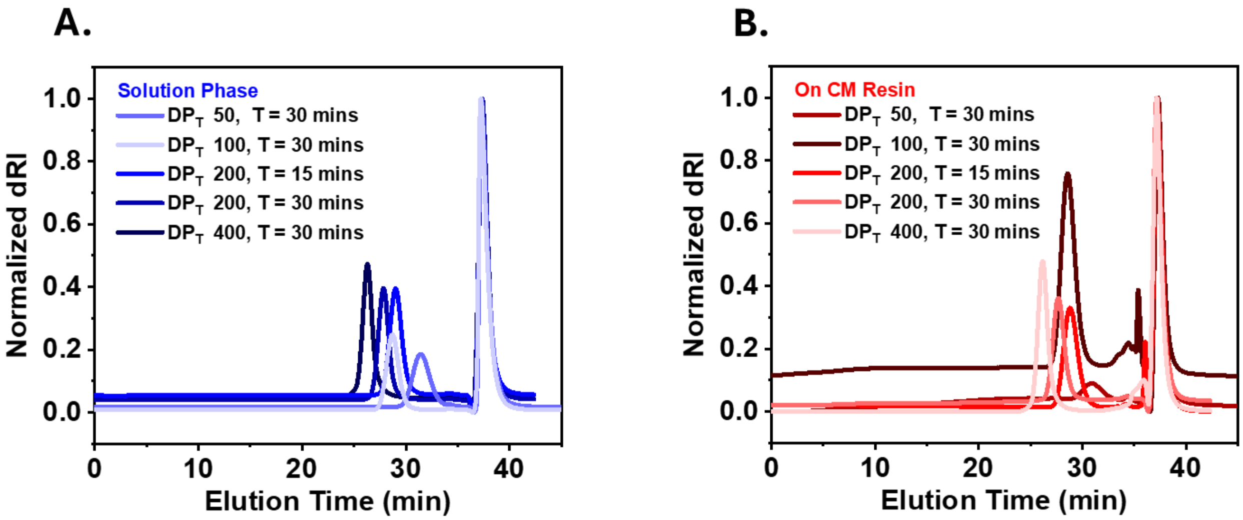

Figure S6. (A) Full SEC Traces of polymers with varying target degrees of polymerization in solution phase and (B) Cleaved from CM resin as shown in Figure 3 (main paper).

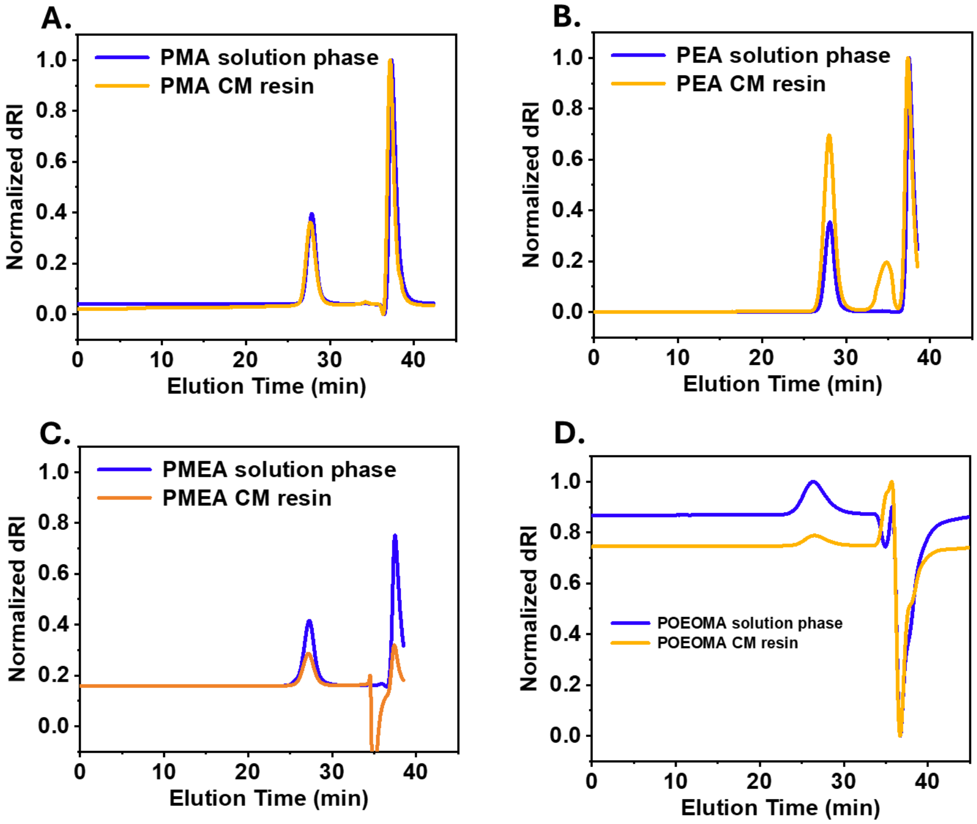

Figure S7. Full SEC traces of polymer samples in Figure 4. (A) PMA, (B) PEA, (C) PMEA, (D) POEOMA.

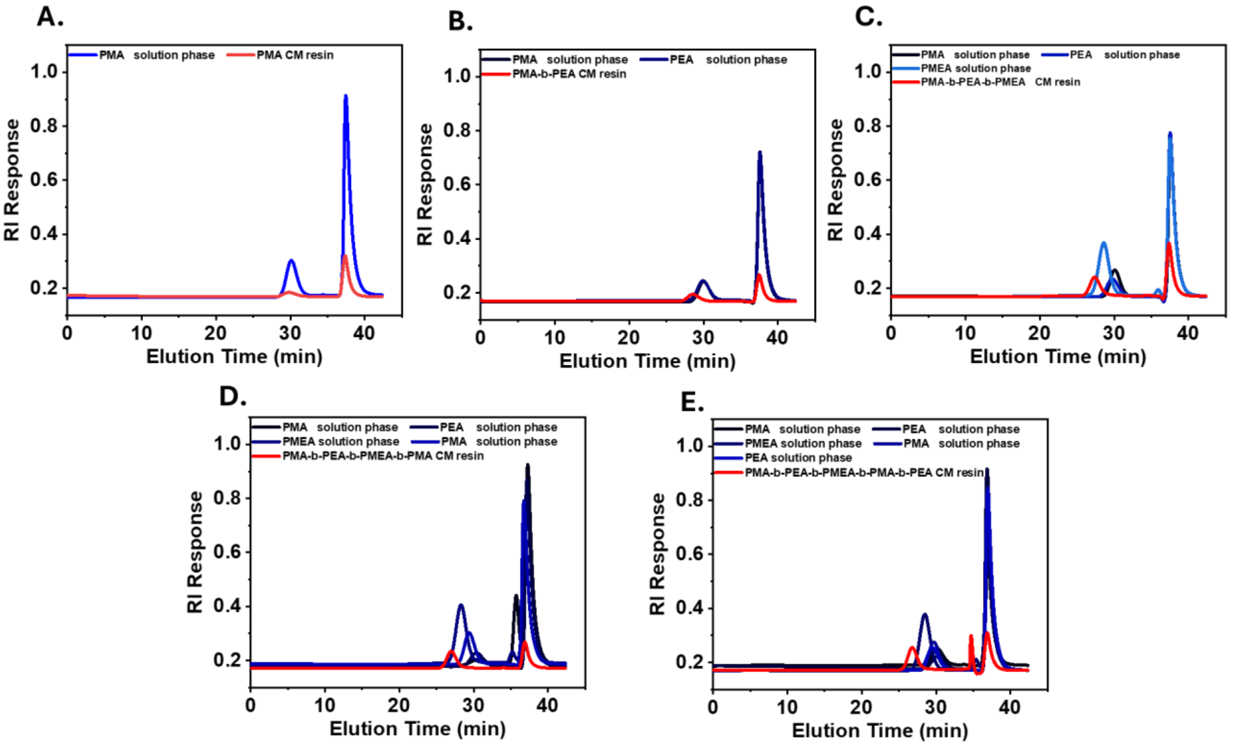

Figure S8. Full SEC traces of multiblock copolymers (A) homopolymer of PMA, (B) diblock copolymer of PMA-*b*-PEA, (C) triblock copolymer comprising of PMA-*b*-PEA-*b*-PMEA,(D) tetrablock copolymer of PMA-*b*-PEA-*b*-PMEA-*b*-PMA, and (E) pentablock copolymer of PMA-*b*-PEA-*b*-PMEA-*b*-PMA-*b*-PEA.

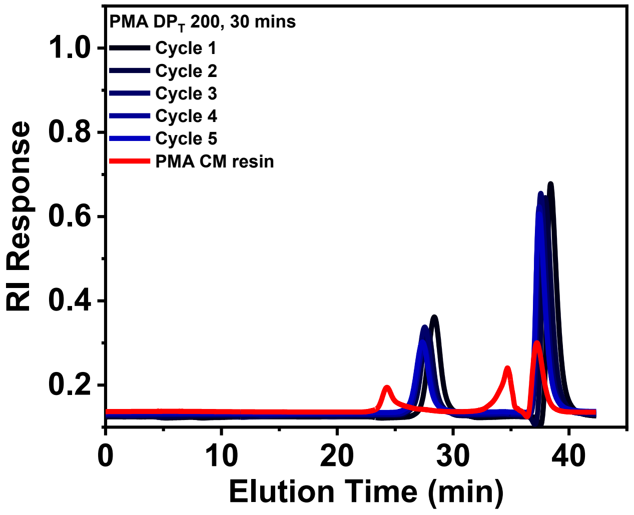

Figure S9. Full SEC traces of polymers in Figure 6.

Synthesis of block copolymers by PICAR ATRP in the solution phase (DMSO)

Synthesis of PMA

The starting stoichiometric condition for the synthesis of PMA was as follows: [MA]/[EBiB]/[$CuBr_2$]/[Me_6TREN]/[SP] = 100/1/0.05/0.15/0.18.

MA (2.5 ml, 27.5 mmol) and DMSO (2.3 mL) were taken into a scintillation vial with a magnetic stirrer. Then, the stock solutions of $CuBr_2$ and Me_6TREN in DMSO and SP in distilled water were introduced into the vial. Finally, EBiB (40.4 µL, 0.275 mmol) was added to the vial, which was placed in the photoreactor under continuous mixing. The reaction environment was exposed to UV irradiation (λ= 390 nm) in open air. After 20 minutes, the mixture was diluted with THF, passed through a short column of neutral alumina, concentrated, and precipitated in a methanol/water mixture (1/1 by volume). The solution phase was decantated, and the polymer was reprecipitated in methanol/water. The material was dissolved in DCM, and the solution phase was dried over $MgSO_4$, filtered off, and taken under vacuum to give PMA ($M_{n,GPC}$ = 9500, $Đ$ = 1.13).

Synthesis of PMA-*b*-PEA

The starting stoichiometric condition for the synthesis of PMA-*b*-PEA was as follows: [EA]/[PMA]/[$CuBr_2$]/[Me_6TREN]/[SP] = 100/1/0.05/0.15/0.18.

PMA (2.12 g, 0.223 mmol) was dissolved in EA (2.38 ml, 22.3 mmol)/DMSO (2.3 mL) mixture thoroughly in a scintillation vial with a magnetic stirrer. Then, the stock solutions of $CuBr_2$ and Me_6TREN in DMSO and SP in distilled water were introduced into the vial. The vial was placed in the photoreactor under continuous mixing. The reaction environment was exposed to UV irradiation (λ = 390 nm) in open air. After 15 minutes, the mixture was diluted with THF, passed through a short column of neutral alumina, concentrated, and precipitated in a methanol/water mixture (1/1 by volume). The solution phase was decantated, and the polymer was reprecipitated in methanol/water. The material was dissolved in DCM, and the solution phase was dried over $MgSO_4$, filtered off, and taken under vacuum to give PMA-*b*-PEA ($M_{n,GPC}$ = 20800, $Đ$ = 1.07).

Synthesis of PMA-*b*-PEA-*b*-PMEA

The starting stoichiometric condition for the synthesis of PMA-*b*-PEA-*b*-PMEA was as follows: [MEA]/[PMA-*b*-PEA]/[$CuBr_2$]/[Me_6TREN]/[SP] = 200/1/0.05/0.15/0.18.

PMA-*b*-PEA (1.31 g, 0.0634 mmol) was dissolved in MEA (1.63 ml, 12.68 mmol)/DMSO (1.6 mL) mixture thoroughly in a scintillation vial with a magnetic stirrer. Then, the stock solutions of $CuBr_2$ and Me_6TREN in DMSO and SP in distilled water were introduced into the vial. The vial was placed in the photoreactor under continuous mixing. The reaction environment was exposed to UV irradiation (λ = 390 nm) in open air. After 15 minutes, the mixture was diluted with THF, passed through a short column of neutral alumina, concentrated, and precipitated in a methanol/water mixture (1/1 by volume). The solution phase was decantated, and the polymer was reprecipitated in methanol/water. The material was dissolved in DCM, and the solution phase was dried over $MgSO_4$, filtered off, and taken under vacuum to give PMA-*b*-PEA-*b*-PMEA ($M_{n,GPC}$ = 37500, $Đ$ = 1.15).

Synthesis of PMA-*b*-PEA-*b*-PMEA-*b*-PMA

The starting stoichiometric condition for the synthesis of PMA-*b*-PEA-*b*-PMEA-*b*-PMA was as follows: [MA]/[PMA-*b*-PEA-*b*-PMEA]/[$CuBr_2$]/[Me_6TREN]/[SP] = 1660/1/0.05/0.15/0.18.

PMA-*b*-PEA-*b*-PMEA (0.5 g, 0.0133 mmol) was dissolved in MA (2 ml, 22.1 mmol)/DMSO (2 mL) mixture thoroughly in a scintillation vial with a magnetic stirrer. Then, the stock solutions of $CuBr_2$ and Me_6TREN in DMSO and SP in distilled water were introduced into the vial. The vial was placed in the photoreactor under continuous mixing. The reaction environment was exposed to UV irradiation (λ = 390 nm) in open air. After 13 minutes, the mixture was diluted with THF, passed through a short column of neutral alumina, concentrated, and precipitated in a methanol/water mixture (1/1 by volume). The solution phase was decantated, and the polymer was reprecipitated in methanol/water. The material was dissolved in DCM, and the solution phase was dried over $MgSO_4$, filtered off, and taken under vacuum to give PMA-*b*-PEA-*b*-PMEA-*b*-PMA ($M_{n,GPC}$ = 110000, $Đ$ = 1.68).

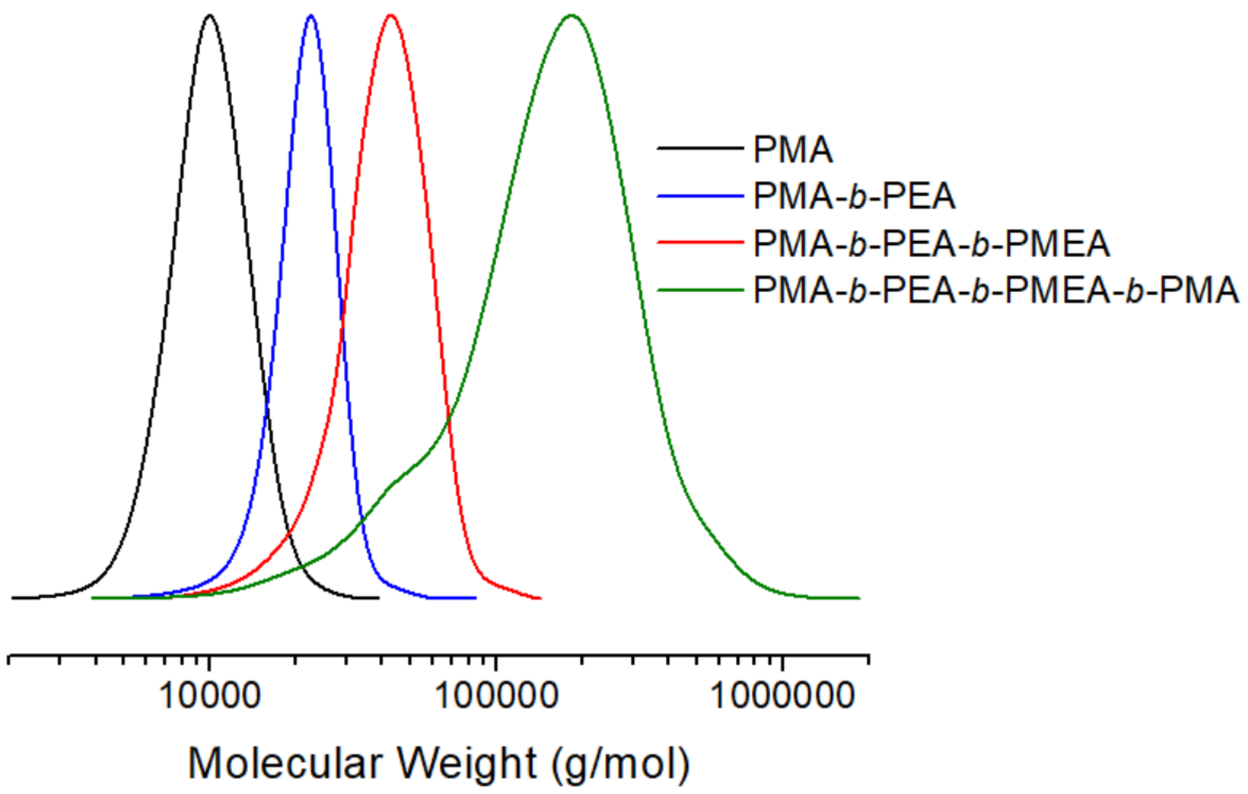

Figure S10. SEC traces of the copolymers prepared by PICAR in DMSO.
